# Voices of Children with Cancer and Their Siblings in the Family Talk Intervention

**DOI:** 10.3390/children12030266

**Published:** 2025-02-21

**Authors:** Maria Ayoub, Malin Lövgren, Ulrika Kreicbergs, Camilla Udo

**Affiliations:** 1 School of Health and Welfare, Dalarna University, 79188 Falun, Sweden; cud@du.se; 2 Department of Health Care Sciences, Palliative Research Centre, Marie Cederschiöld University, 11628 Stockholm, Sweden; malin.lovgren@mchs.se (M.L.); u.kreicbergs@ucl.ac.uk (U.K.); 3 Advanced Pediatric Home Care, Astrid Lindgren Children’s Hospital, Karolinska University Hospital, 17164 Stockholm, Sweden; 4 Louis Dundas Center, Great Ormond Street Institute of Child Health, University College London, London WC1E 6BT, UK; 5 Department of Women’s and Children’s Health, Childhood Cancer Research Unit, Karolinska Institute, 17177 Stockholm, Sweden

**Keywords:** child participation, Article 12, psychosocial support, family-centered, pediatric oncology

## Abstract

Background: Children in pediatric oncology report unmet needs related to communication and information about the illness, care involvement, and psychosocial support. Supporting the whole family involves challenges, with a risk that children’s voices are not heard above those of the adults. Article 12 of the UNCRC has been a catalyst in supporting children’s voices and their right to participate in processes that affect them. The aim of this study was to explore how children with cancer and their siblings experienced participation in a family-centered psychosocial support intervention, the Family Talk Intervention (FTI). Methods: Interviews were held with 35 children (ill and siblings) from 26 families in pediatric oncology after having completed the FTI. A combined deductive and inductive qualitative content analysis was undertaken, guided by the Lundy model of child participation. Results: Children’s experiences of being able to express their views, being listened to, and being involved during FTI were mainly positive. This was related to their participation in individual meetings where they could raise their concerns and views, undertake small activities while talking, and have their voices and needs mediated to relevant adults, such as parents and professionals. Conclusions: The findings of this study showed that the FTI for families in pediatric oncology created opportunities to promote child participation. These findings indicate that, by offering children an individual space where they can express themselves freely and supporting them in various ways to do so, the children’s voices and involvement are strengthened.

## 1. Introduction

When a child is diagnosed with a life-threatening illness such as cancer, this has psychosocial consequences for the child, their siblings, and their parents [1,2]. Current recommendations state that pediatric oncology units should offer psychosocial support to the whole family alongside the child’s medical treatment and care [3,4,5]. In addition, children should be supported and encouraged to participate in matters that affect them [6]. However, children in pediatric oncology have reported unmet needs related to information about the illness, communication, care involvement, and access to psychosocial support [7,8]. This can contribute to feelings of powerlessness, fear, and worry, which can increase the risk of psychosocial problems for children with cancer and their siblings [9,10,11].

Psychosocial support interventions, i.e., the provision of psychological, spiritual, and social support [12], are crucial to helping families affected by childhood cancer [2,13,14]. An evaluation of a few such interventions, targeting the whole family, has shown improvements in the wellbeing of all family members, for example, through reduced distress, decreased anxiety and depression, and improved quality of life [15,16]. Effective psychosocial support interventions for children with cancer and their siblings have been found to include those promoting autonomy and involvement [11,17].

Supporting the entire family when a child has a severe illness is based on the belief that children’s wellbeing is best achieved when healthcare professionals support the parents’ ability to meet their child’s needs [18]. However, although the intention of family interventions is to involve all family members, there is a risk that the child’s perspective is not heard above the voices of the adults [19,20].

Children in healthcare services have rights under the United Nations Convention on the Rights of the Child (UNCRC), specifically Article 12, regarding participation in processes that affect them and being listened to [21]. Since Article 12 does not provide a definition of children’s participation or how to operationalize this, the UN Committee on the Rights of the Child [22] has, in General Comment No. 12, defined it as a process including mutual respect between children and adults, information-sharing, and where children are made aware of how their views are taken into account. Although the UNCRC has been adopted in policies worldwide, challenges remain in actually implementing the principles [23,24]; the child’s views are not always given due weight [21,25]. In Sweden, in addition to the UNCRC, an ill child’s position is further strengthened by the Health Care Act [26] and the Patient Act [27]; however, the position of siblings is not explicitly stated.

One psychosocial support intervention that includes parents, the child with severe illness, and their siblings is the Family Talk Intervention (FTI) [28]. The FTI was originally developed for psychiatric care to target families with dependent children where a parent has depression. The aim of FTI is to increase open and child-centered family communication about the illness and related subjects, support parenting, and build resilience. The children should be central throughout the FTI, with a focus on making their needs visible and their voices heard [29]. In psychiatric care, evaluations of FTI have shown enhanced family and child psychosocial functioning, including improved family communication and relations, and increased illness-related knowledge [30,31]. Recently, FTI has been used in somatic care, where a parent is affected by a severe illness, with promising results [32].

While family-centered interventions have their central focus on the children, they still risk highlighting the adults’ perspectives and wishes while not fully prioritizing the children’s needs [20,33]. For instance, in an evaluation of children’s participation in FTIs in palliative care when a parent has a severe illness, it was found that children’s views were not considered to the extent wished for [20]. This is despite the main focus of the FTI, where the children should be involved in deciding the family’s goals for the FTI and the topics to be raised [29]. Although the involvement of children is central in family-centered psychosocial support interventions, such as the FTI, few if any have analyzed whether and how children are involved in the practices and how they view their participation.

### Aim

The aim of this study is to explore how children with cancer and their siblings experienced participation in the FTI.

## 2. Theoretical Framework

Various models have been developed to conceptualize child participation. One of the most widely known models, central to this study, is the Lundy model of participation [34]. This focuses on child participation from a rights perspective and provides a conceptualization of a child’s right to participate, as stated in Article 12, recognizing children’s involvement as an ongoing dynamic process. The model has previously proven useful in exploring child participation in healthcare contexts in accordance with Article 12 [21,25]. It states that the successful implementation of Article 12 requires consideration of four interrelated key elements that embody children’s rights to express their views that should be given due weight: space, voice, audience, and influence. The interrelatedness of the elements acknowledges the overlap between space and voice, closely linked to the first part of Article 12 (right to express a view), and between audience and influence, linked to the second part of the article (right for their views to be given due weight).

The element space requires children to be given the opportunity to express a view in a safe and inclusive space. Voice means that children are allowed to express their views freely regardless of their age or maturity. Audience requires that the children’s views are actively listened to by those who can give effect to the children’s views. Influence is described as ensuring that the children’s views are given due weight and acted upon appropriately.

## 3. Materials and Methods

### 3.1. Design

This study derives from a pilot intervention study of the FTI in pediatric oncology with a focus on all family members. It uses mixed methods with a pretest–posttest design [35]. The present study has a qualitative descriptive design. A combined deductive and inductive analysis was conducted using the Lundy model [34] as a conceptual framework to guide the analysis, focusing on the children’s (ill child and sibling) descriptions.

### 3.2. Study Setting and Participants

Children and their families were recruited from one pediatric oncology center in Sweden over a one-year period (September 2018 to August 2019), two to three months after the child’s diagnosis or relapse. Eligible families were Swedish-speaking and had at least one child aged 6–19 years (ill child or sibling). The whole, or part of, the family could participate, with a minimum of one parent and one child aged 6–19 years per family. Nurses at the center identified families matching the inclusion criteria. Each family was then contacted by phone by an interventionist, i.e., an FTI-educated clinician (two social workers and one deacon who worked in pairs), to arrange a meeting where all family members received age-appropriate verbal and written information about the study. Participating parents and children over 15 years gave written informed consent, in accordance with Swedish law. Written informed consent was obtained from parents/guardians for children under 15 years. Although the parents provided legal permission for their children to participate in the study, the children (ill or sibling) gave signed consent to be interviewed. Of the 61 eligible families, 27 consented to participate and 26, with a total of 63 children (ill children, *n =* 26; siblings, *n =* 37), completed the intervention. In this study, interview data from 35 children (ill children, *n =* 10; siblings, *n =* 25), based on their age, were included (Table 1).

### 3.3. The Psychosocial Support Intervention FTI

The core elements of the FTI are to facilitate family communication, increase family members’ understanding of the illness, support parenting, and help the families to identify their strengths and how to use them best. The FTI is built upon an eclectic approach that includes narrative, dialogical, and psychoeducative ways of working. Through the narrative and dialogical ways of working, family members share their experiences and stories and thereby create a shared understanding of the situation by making each other’s perspectives and needs visible. The psychoeducative part focuses on increasing knowledge about the illness [36,37].

FTI is manual-based and involves 6–11 meetings at intervals of 1–2 weeks with family members in various constellations (Table 2). The FTI manual is a structured guide that provides an outline for each meeting, containing different focus areas and topics for discussion. Since the FTI had not yet been evaluated in a pediatric care context, the manual was used in the same way as in adult care, with one exception. To consider children’s perspectives and participation to a greater extent, the children’s relationships with each other in the family were incorporated into the original manual.

The meetings were led by FTI-educated professionals, in this study referred to as interventionists, who worked in pairs, in a place chosen by the family, often in their home.

### 3.4. Data Collection

This study focuses on semi-structured interviews with the children, both the ill child and their siblings, aged six years and older. The interviews were conducted by members of the research team who accommodated the children’s wishes concerning where the interview was performed and if they wanted to be interviewed in pairs or alone. All interviews were held in the children’s homes. The interviews were guided by a semi-structured interview guide focusing on the children’s views about the FTI and their overall experiences of participating, for example, regarding timing, what they perceived as being good or bad within the FTI, the opportunity to express wishes for specific support or help, and whether they had been listened to. Although questions were not asked explicitly about the children’s views on expressing their views and having them heard during FTI, information about this emerged. They were, for example, asked about their experiences of the individual meetings with the interventionists and the family meeting.

The ill children were interviewed alone except for one ill child who was interviewed in the presence of a family member, and two siblings who were interviewed with another sibling. The interviews lasted between 7 and 36 min and were audio-recorded and transcribed verbatim.

### 3.5. Data Analysis

The interview transcripts were analyzed using combined deductive and inductive content analysis [38,39], starting deductively. Since the analysis was to be based on the children’s experiences as precisely as possible, the focus was on the manifest content. A categorization matrix was developed in accordance with the Lundy model in which the data were reviewed, sorted, and analyzed based on the four key elements of space, voice, audience, and influence (Table 3).

In the deductive phase, each interview was read in its entirety to gain a sense of the whole. Text segments that, on first impression, appeared to represent elements of participation, for example, having a safe space or being listened to, were then highlighted by the first author. The segments corresponding to the categorization matrix were then coded and transferred into the relevant elements based on Lundy’s [34] description of each key element.

In the next phase, the key elements were analyzed inductively one by one. The analysis began with repeated readings of the transferred text segments for each key element to gain an in-depth understanding, beyond the earlier categorization of the text in the deductive phase. Additional text segments were identified and condensed within the key elements. Those text segments describing the same thing were grouped and given a code. The codes were abstracted and sorted into sub-elements to gain further understanding of the children’s experiences of participating in FTI as derived from the matrix.

The primary analysis was conducted by the first author and was continuously discussed with the last author before all co-authors discussed the findings and a consensus was reached. There is always more than one possible interpretation of the text; therefore, the interpretations and elements were discussed and reflected upon by all authors, resulting in the presented findings representing the most credible understanding of the data. Furthermore, in order to enhance credibility and trustworthiness, examples of verbatim quotations are presented.

### 3.6. Ethical Considerations

This study received ethical approval from the Regional Ethical Review Board in Stockholm (No. 2018/250-31/2 and 2018/1852-32) and was conducted in accordance with the Helsinki Declaration [40]. Information about the study was simplified to ensure that the children could understand the goal. The children were informed about the risks and advantages of the study, that their participation was voluntary and could be withdrawn at any time, and that the research data would be treated as strictly confidential. Those who were willing to participate were asked to provide informed consent. Written consent was obtained from parents/guardians for children under 15 years, while children over 15 years gave consent for their participation according to Swedish law [41,42].

## 4. Results

The results are presented according to the four key elements in the Lundy model of participation [34]: space, voice, audience, and influence. Sub-elements related to these are also presented (Table 4). In the text below, children’s participation in the FTI is presented within the four key elements and illustrated with verbatim quotations from the interviews.

### 4.1. Space

The sub-elements identified for space were safe place, comfort, and trustful relationship.

The children described how the individual meetings with the interventionists created a safe place in which they felt comfortable and able to talk freely and express their views and concerns. According to the children, these meetings provided an opportunity to talk about whatever was important to them in their current life situation.

They also reported talking to the interventionist about things they did not tell their parents for fear of hurting or worrying them. They explained that the individual meetings offered a space where they could ask questions about the illness that they thought their parents would not be able to answer. The opportunity to talk undisturbed to someone outside the family was described as enabling an openness where they could raise their thoughts and emotions, whatever these concerned.

*I think it was really good to meet them [interventionists] and talk to someone outside the situation […] Because I was just walking around trying to survive each day, and then when I met them [interventionists] I could cry, I could do everything, tell them everything*

(Sibling)

Although most children described being given the opportunity to share their views, they sometimes felt uncomfortable talking to the interventionists, particularly when meeting them for the first time.

*I thought it was a bit difficult at the beginning. Because it took quite a long time for me to get to know them and we only met them [interventionists] twice, or I only met them twice, I didn’t get to know them very well*

(Sibling)

In addition, because the support intervention was limited, some children were uncertain about what and how much they should share despite having the space to do so. The children described how being interviewed in their own home made them feel comfortable and relaxed. One child explained that having their sibling present helped them feel more secure and able to express their views. There were times, however, when the children did not want to express their opinions. In such situations, the children described having the opportunity to refrain, take a break, or end the meeting.

### 4.2. Voice

The sub-elements identified for voice were being asked questions, their views being mediated, and being able to share ideas.

The children reported that the interventionists, in various ways, provided them with the necessary support to express their views during the FTI, for example, by engaging in activities during the conversation, such as drinking tea or playing with slime, and by asking them questions. The children described how the questions helped them to communicate their perspectives, feelings, and wishes. However, they sometimes experienced being asked too many questions and the interventionists continuing to ask them questions even when they had nothing to say.

The children described how during the individual meetings, they reflected on, together with the interventionists, how to best express their views and thoughts to their siblings and their parents: *“they [interventionists] helped us collect our thoughts so that we were able to talk about them. So we could solve some of the problems that we had in the family”* (Sibling).

In general, the children wanted the interventionists to mediate their views and perceptions to parents and siblings, either in the upcoming family meeting or by talking to them directly. This could include helping them to express themselves during the family meeting or speaking on their behalf. The children also stated that they were encouraged to express wishes and ideas related to family life, such as wanting to spend more time alone with their parents but also more time with the whole family.

The children perceived having the opportunity to contribute to the family meeting, either by speaking for themselves or having the interventionists speak for them and were able to share their thoughts and feelings with the family. However, sometimes children found it difficult to express themselves when the whole family was gathered.

### 4.3. Audience

The sub-elements identified for audience were being listened to (directly/indirectly), being respected, and being relevant.

During the FTI, the children experienced having someone to communicate their views to, if they wanted, who was in a position to give effect to their views. Children often described that the interventionists listened to them and respected their viewpoints. One child reported asking the interventionists to help them receive more information about the illness, although not too detailed. The interventionists had actively listened to these wishes and respected the amount of information wanted. Furthermore, the children described sometimes raising topics that were not directly related to the illness but instead to their everyday lives, which the interventionists listened to. However, sometimes they perceived that the interventionists did not really pay attention to topics they considered meaningful, for example, related to their hobbies, but instead moved the conversation on.

In the meetings, the children described being informed about and becoming aware of family members’ views as mediated by the interventionists.

*in the children’s conversation, my little sister said she thought that I didn’t have friends at school […] then later in the family conversation, they [interventionists] brought it up, and then we talked about it. I think it felt good to talk about it*

(Sibling)

In some cases, with approval from the child concerned, the interventionists provided the children with information about their siblings’ worries and needs, as illustrated in the quotation above. Information about how a sibling or parent perceived their situation was described to help the children feel more involved in the family.

Although the children mainly reported being listened to, sometimes the child perceived that the interventionists listened primarily to the parents and to their views and perspectives.

In other cases, the children experienced that their views, needs, and wishes were more likely to be listened to and respected when first relayed to the interventionists in the individual meetings who then communicated them to other adults, mainly professionals such as physicians or teachers. The interventionists were then described by the children as a link between them and others, such as professionals or family members.

*I think I spoke to her [interventionist] straight away. And then I think... she asked if it was okay to tell Dad. And it was, so I think that’s why...He [father] listened a bit more when someone else said it*

(Child with cancer)

Not being listened to by their teachers made it difficult for the children to continue trying to communicate with them. When the interventionists listened to how things were at school, they could then make the teachers aware by helping to provide the school with information about the home situation.

### 4.4. Influence

The sub-elements identified for influence were involvement, views being acted upon, and being taken seriously.

During the FTI, the children experienced being somewhat involved in matters that affected them. They described their possibilities to tailor the individual meetings with the interventionists to their preferences and needs. Further examples of influence were, e.g., when their voices were acted upon by the interventionists and their parents. One child explained that after having expressed a desire to be continuously updated about the sibling’s treatment, they had become more included in the information process and felt more involved in their sibling’s healthcare. Other children described how their views had been taken seriously and acted upon, primarily concerning school issues. For example, in decisions regarding school support, children described shared efforts being made by the interventionists and their parents to give their views due weight.

*I didn’t want to go [to school], so then me and mom went for coffee, and I could tell her why […] It really didn’t feel good being there, and so mom called my teacher, and then I had to take a lot of stuff home, and I had teachers at the hospital. And some days my teachers came to my house...*

(Child with cancer)

The children described how their views were taken seriously and mostly acted upon by their families. In most families, the children reported that, after raising their wishes, they were involved in the negotiation of family rules and in deciding on and carrying out activities in the family, e.g., regular family meetings, eating dinner together, adopting healthier habits, and spending time alone with their parents. However, some children also experienced their views and wishes not being taken seriously by their families.

*When everyone in the family was at home, we shouldn’t sit with our mobile phones. Instead you should go up to your room and do it […] I don’t think that’s really being followed. There are still some people sitting down there with their mobile phones*

(Child with cancer)

Even though the interventionists advised the parents to take their children’s views and suggestions seriously and be open to being influenced by them, some children experienced having little real influence on decisions made within the family. They reported that the opinions and suggestions they raised during the family meeting were not always taken into account afterward.

## 5. Discussion

This is one of few studies exploring what children say about taking part in a family-centered psychosocial support intervention, the FTI, in pediatric oncology. Children’s participation, i.e., their subjective experiences of involvement, was explored using the Lundy model, which outlines that a child’s right to participate, in accordance with Article 12 of the UNCRC, is achieved through the realization of the key elements of space, voice, audience, and influence. The overall findings indicated that the FTI, despite starting with a meeting with the parents, created opportunities to reach all the aspects of child participation according to Article 12 of the UNCRC. Nevertheless, the children also made concrete suggestions for how to further enable children’s participation.

In this study, children described overall positive experiences in relation to the element of space. The findings showed that the creation of a safe space, where the professional and the child can meet, is a prerequisite, which is in line with previous studies on how to accomplish child participation within healthcare and social services [43,44]. The individual meetings with the interventionists were held in the children’s homes, spaces where the children generally felt comfortable sharing their views, thoughts, and concerns about their current life situation. According to the children, trusting the person providing the psychosocial support is essential to creating a safe space. Positive relationships with professionals have previously been described as contributing to children feeling more confident in expressing a viewpoint [45,46]. Furthermore, the children in this study also suggested meeting the interventionists once before the individual meeting to establish a relationship and thus create a safe space, a suggestion that might enable a child to express their voice in healthcare and social services.

In relation to the key element of voice, the children’s descriptions were mostly positive. They appreciated being allowed to undertake small activities while talking with the interventionists, i.e., playing or drinking something, a well-reported strategy to help children feel confident in communicating their thoughts, feelings, and views in healthcare and social services [47]. Being asked questions by the interventionists was another important element in facilitating the expression of children’s voices during FTI. Based on the children’s descriptions, professionals need to be sensitive, for example by balancing the number of questions, so that children do not feel pressured to express their views but instead invited to do so. Inviting children by asking questions could be used to confirm that their views are expressed and show that their views matter [48]. Moreover, the children reported that they often received support from the interventionists to mediate their thoughts, wishes, and views to other family members. This indicates the responsibility that adults have to help children express their views, either by facilitating the children to speak for themselves or by representing their views [45]. Although children are central and their participation is often an explicit goal in family-centered psychosocial interventions such as FTI [28], sometimes children may not want to express a viewpoint or even participate [43], as evidenced in this study. Participation according to Article 12 of the UNCRC is a right and not an obligation, meaning that children are entitled to choose whether or not to express a view [34].

During the family meeting, the children sometimes described limited opportunities for expressing their views. These findings support the existing literature highlighting this as a risk with a family-centered approach [49], where children’s views and voices co-exist with those of others [50]. Studies have indicated that children experience difficulties in expressing their views freely when their parents are present in meetings within healthcare or social services, something that may potentially impede child participation [51].

However, the children’s overall experiences of space and voice in this study indicate that the right for children to express their views has been considered during the FTI.

In relation to the element of audience, children described both positive and negative experiences. Having an engaged audience is one important prerequisite for children’s voices to be heard [52], also highlighted in this study. According to the findings, children wished for and received tailored illness-related information, indicating that the children’s views were listened to and respected by the interventionists during FTI. This supports other studies that highlight the importance of children with cancer and their siblings in pediatric oncology receiving age-adapted information about the illness in order to feel involved [53,54]. Similar to previous studies [55] and in relation to the element of audience, children in this study described being heard to a greater extent when their views were presented by the interventionists to other adult professionals, such as teachers. The provision of psychosocial support to families in crisis often includes sharing advice with parents on how best to support their children, which is one of the main goals of the FTI [29]. This could also include identifying and referring children to appropriate services. In such situations, healthcare professionals are in a position to serve as a bridge to schools and other child and family services [56]. Several professionals and organizations might therefore be involved as relevant audiences for the child’s voice [57].

Similar to children’s experiences of the element of voice, their descriptions of influence were both positive and negative. As previously shown, children’s feelings of being heard and that their views matter are central to their experience of participation [34,44], which was evident in this study. Even though the structure of the FTI starts with asking the parents about the situation, the children in this study mostly experienced that the FTI meetings were tailored to their individual preferences and needs. During participation in the FTI, the children felt that their expressed needs and wishes, mostly related to school issues, were listened to and taken seriously by the interventionists and acted upon by their parents. This is in line with other studies, showing that children’s involvement in healthcare and social services benefits from the shared efforts of professionals, children, and their families [43], which also is facilitated within the FTI. Following up on whether and how the children’s expressed views and preferences were given due weight and had influence is another important aspect of children’s participation within healthcare and social settings [58]. In this study, children reported that their wishes and suggestions were not always considered by the family. This would suggest including more feedback or follow-up regarding actions taken after listening to the children’s views during the FTI.

While family interventions in healthcare contexts suggest that the child is at the center, parents’ voices may, however, take priority [19]. This creates challenges for healthcare professionals to both acknowledge parental responsibility and simultaneously recognize the participatory rights of children [59]. Although the original structure of the FTI, with only one individual meeting per child, may not in itself promote child participation, the findings in this study indicated that the children could express their views freely and influence how the information was handled and whether it was communicated to their parents. Furthermore, the situations in this study, described by the children, indicate that participation is a process of reciprocity. This is consistent with the assumption that child participation is a mutual process meaning that children, like adults, can also have a voice, listen, and act [60]. For instance, during the FTI, the children both had an audience for their voices and were the audience when listening to their family members.

This study contributes to the suggested need to include children, as a vulnerable but competent group, in research in order to access their voices, perspectives, and experiences [61]. However, this study has some limitations. The interventionists involved might have been particularly proficient in facilitating children to express their views and be heard by other relevant adults. Other limitations are related to the representativeness of the findings. As a qualitative study, the results are not generalizable. Also, the findings are based on interviews with children from only Swedish-speaking families at one pediatric oncology center. A more sensitive consideration of the social and cultural context of children is therefore necessary for an understanding of child participation in family-centered psychosocial support interventions. Including both children with cancer and their siblings can be a limitation since their experiences of participation in the FTI may differ. However, the structure of the FTI does not focus on or distinguish between whether one participates as a sibling or as a child with cancer, which strengthens the inclusion of all children in this study. As with all qualitative interview studies, the interviews in this study rely on the participant’s ability to accurately recall details about their experiences. Since the children were between 6 and 19 years old, they had different developmental levels and communication skills, which also may have affected the interviews. However, this was not apparent in relation to their experiences of participation in the FTI. Another limitation is the length of the interviews; however, although they lasted for a relatively short time, the children’s experiences of their participation in the FTI were captured.

This study could contribute to a more complete picture of how child participation could be put into practice, provide insights into what considerations and actions can be taken to ensure that children’s voices are heard and taken into consideration in family-centered interventions, and thus improve child participation. The FTI has the potential to make the children’s voices heard, both ill children and siblings, and help them manage the difficult situation when living with childhood cancer. Thus, when implemented and evaluated in clinical practice, the FTI could be a concrete way of working for healthcare professionals when supporting these families. However, in future studies on the FTI, the child’s perspective could be strengthened further in FTI education, as well as in the FTI manual to increase the children’s participation and their rights according to Article 12 of the UNCRC.

## 6. Conclusions

The findings in this study suggest that each element of a child’s right to participate, in accordance with Article 12 of the UNCRC, does not occur separately in the FTI but as part of a reciprocal process involving all four elements of space, voice, audience, and influence. This study indicates that by offering the child a safe space where they can express themselves and supporting the child in reaching the intended audience, the child’s voice and influence are strengthened. The findings further suggest that individual meetings with each child facilitate the child’s participation in a family-centered psychosocial support intervention.

## Figures and Tables

**Table 1:** Demographic information on participating families and interviewed children.

Numbers of Families Participating in FTI, *n* = 26			Children Taking Part in an Interview	
	Children with cancer, *n* = 26 *N* (%)	Siblings, *n* = 37	Children with cancer, *n* = 10 *N* (%)	Siblings, *n* = 25
**Age (years)**				
Mean age	10	11	11	10
Min-max	1–17	3–24	7–17	6–23
**Sex**				
Female	14 (54)	20 (54)	6 (60)	16 (64)
Male	12 (46)	17 (46)	4 (40)	9 (36)
**Cancer diagnosis**				
Central nervous system tumor	13 (50)		4 (40)	
Leukemia	4 (15)		3 (30)	
Lymphoma	5 (19)		1 (10)	
Sarcoma	2 (8)		1 (10)	
Other	2 (8)		1 (10)	

**Table 2:** Contents of each meeting in FTI and the family members involved.

Meeting	Family Members Involved	Contents
1–2	Parents	The parents’ stories and experiences of the situation. The parents’ concerns about the children and wishes regarding topics and questions to raise with the children. Parents’ wishes regarding family goals to achieve during FTI.
3	Each child	The child’s story and experience of the situation, worries, and questions. The child’s wishes regarding topics and questions to raise and goals to achieve during FTI.
4	Parents	Summary of the children’s worries, questions, and wishes raised during Meeting 3. Planning ‘the family talk’ (Meeting 5).
5	Parents and children	‘The family talk’. Led by the interventionists and covering topics and issues raised by both children and parents.
6	Parents and sometimes children	Follow-up with a focus on how to communicate within the family in the future to achieve the family’s goals.
7		Extra meetings if needed.

**Table 3:** Categorization matrix.

	Space	Voice	Audience	Influence
Child participation in a psychosocial support intervention	Children are given opportunities to express their views in a safe environment, in which they feel comfortable.	Children are facilitated in forming and expressing their views. Children are provided with appropriate information, support, and guidance from the interventionists so they can form a view.	Children’s views are actively listened to and respected. Children are guaranteed opportunities for their views to reach those audiences who have the responsibility to listen.	Children’s views are taken seriously and acted upon appropriately. Children are involved in the process of FTI and feel there is an openness to being influenced by their views.

**Table 4:** Sub-elements deriving from the key elements of the Lundy model.

Key Elements	Space	Voice	Audience	Influence
Sub-elements	Safe place	Being asked questions	Being listened to (directly/indirectly)	Involvement
	Comfort	Views being mediated	Respected	Views being acted upon
	Trustful relationship	Sharing ideas	Relevant individuals	Being taken seriously

## Data Availability

Data are available upon request due to privacy and ethical restrictions.
